# Prognosis of COVID-19 in the middle eastern population, knowns and unknowns

**DOI:** 10.3389/fmicb.2022.974205

**Published:** 2022-08-31

**Authors:** Iman Dandachi, Waleed Aljabr

**Affiliations:** Research Center, King Fahad Medical City, Riyadh, Saudi Arabia

**Keywords:** SARS-CoV-2, diabetes, cancer, children, pregnancy

## Abstract

Since its emergence in China in 2019, the SARS-CoV-2 virus has affected all countries worldwide. The virus is easily transmitted from one person to another *via* infected aerosols or contaminated surfaces. Unlike its counterparts, the prognosis of COVID-19 ranges from asymptomatic to critical disease or death. Several factors play a role in determining the severity of the disease in infected patients. Among others, is the pre-existence of an underlying medical condition such as diabetes, cancer, and others. Furthermore, although children are less prone to the severe form of the COVID-19 disease, they require attention due to the report of many atypical presentations of the infection, post-asymptomatic exposure. In the Middle East, little is known about the prognosis of the SARS-CoV-2 infection in high-risk categories, notably patients with diabetes, cancer, and pregnant women. The aim of this review is to summarize the current knowledge about this group of population in the middle eastern region as well as to highlight the gap in the literature. We have found that the majority of the papers were from the Gulf countries. Although, few studies were conducted; high-risk patients appear to have an increased risk of morbidity and mortality from COVID-19 compared to their counterparts. Higher levels of inflammatory markers, C-reactive protein, erythrocyte sedimentation rate, D-dimer, and ferritin levels were also observed. Children are often asymptomatic or present with atypical presentations. More studies should be conducted to determine the clinical biomarkers of COVID-19 in high-risk categories to help in patient risk stratification and management in the middle eastern population.

## Introduction

Coronaviruses (CoVs) are positive single-stranded RNA viruses that have large spike protein molecules on their surface giving them a “crown-like” shape (Pellett et al., 2014). The subfamily of the Coronaviridae family has been classified into four genera: alpha, beta, delta, and gamma (Cascella et al., 2022). Within these, seven are known to infect humans: NL63 and 229E from the alpha-CoV and HKU1, OC43, SARS-COV, MERS-COV, and SARS-CoV-2 from the betaCoV. SARS-CoV-2 is a novel coronavirus that shares around 80% nucleotide sequence similarity with SARS-CoV (Abdelrahman et al., 2020). Following its first detection in China, in 2019, SARS-CoV-2 became a worldwide pandemic that up to 13 April 2022, affected 499,119,316 cases and resulted in 6,185,242 deaths (World Health Organization, 2022b). Compared to SARS-COV and MERS-COV, SARS-CoV-2 appears to be more infectious and contagious (Abdelrahman et al., 2020). During the pandemic, multiple variants have been described. Among these, few were characterized by the WHO as “variants of concerns” (VOCs) due to their either enhanced transmissibility, increased virulence, or decreased effectiveness of the current diagnostics, therapeutics, and vaccines (World Health Organization, 2022a). To date, five VOCs have been identified: Alpha (B.1.1.7), first described in the United Kingdom in late December, Beta (B.1.351) first reported in South Africa in December 2020, Delta (B.1.617.2) identified in India in December in 2020, Gamma (P.1), identified in Brazil in January in 2021, and Omicron (B.1.1.529) reported in South Africa in November 2021 (Cascella et al., 2022). Healthcare workers (HCW) were at the frontlines to confront and combat this pandemic. Between January 2020 and May 2021, the WHO estimated a 115,500 HCWs’ death from COVID-19 (WHO departmental news, 2021). As of April 2020, in Italy, around 10,000 HCWs got infected including 74 that have died (Chersich et al., 2020).

The COVID-19 disease ranges from asymptomatic to pauci-symptomatic to severe illness (Cascella et al., 2022). The center of disease and prevention center mentioned that people with certain medical conditions such as diabetes, cancer, and pregnant women have increased risk of being “very sick” from SARS-CoV-2 infection. This means that an infected person will be more likely hospitalized, admitted to an intensive care unit (ICU), require ventilation to assist in breathing, or in the worst-case scenario die (Center for Disease Control and Prevention, 2022). SARS-CoV-2 can infect all ages including those under 18 years of age and neonates. Compared to adults, the clinical course is often milder and asymptomatic in children; however, it has been reported that this group is more susceptible to co-infection, and can present with atypical symptoms making thus diagnosis of COVID-19 more challenging (Li et al., 2020; Ben-Shimol et al., 2021; Shahin et al., 2021).

In the Middle East, in adults, the first confirmed cases of SARS-CoV-2 were in the United Arab Emirates (UAE) on the 29th of January 2020, followed by Lebanon in 21 February and Bahrain, Kuwait, Oman, and Iraq in the 24th of February of the same year (Alwahaibi et al., 2021). In infants, the first case was reported in Lebanon early in the year 2020 (Mansour et al., 2020). The Middle East is a sensitive area culturally, politically, and economically. Over the past few years, the region was subject to continuous population mobilization in view of the multiple war crisis as well as socio-economic conflicts. This region includes 14 countries: Egypt, Iran, United Arab Emirates, Kingdom of Saudi Arabia (KSA), Qatar, Kuwait, Bahrain, Sultanate of Oman, Yemen, Lebanon, Jordan, Palestine, and Syria. The aim of this review is to summarize the current knowledge about the clinical impact of COVID-19 on healthcare workers, adults with underlying medical conditions as well as younger ages in this distinct area of the world.

## Healthcare workers

### Prognosis and source of infection

Healthcare workers are the building blocks of the healthcare system. According to the WHO definition, healthcare workers are all people engaged in the process of enhancing health. These include nurses, midwives, physicians, paramedical staff but also support staff, hospital managers, and community workers (El-Raey et al., 2021). During the SARS-CoV-2 pandemic, HCW had to work for many hours, over many shifts, at maximum capacity, and in some settings with limited protection (The Lancet, 2020). This made them at increased risk of contracting the infection. During their work, HCWs face excessive exhaustion, psychological distress due to separation from family, own risk of exposure and infection, and risk of introducing the infection to colleagues, family members, and hospitalized patients (Chersich et al., 2020).

In Egypt, studies have shown that most of the infected HCWs are nurses and physicians (Abou-ElWafa et al., 2021; El-Sokkary et al., 2021; Mostafa et al., 2021). This could be due to work overload, extended use/re-use, or suboptimal use of personal protective equipment (PPE), and the transmission of the virus from a HCW to another. Environmental factors can also play a role, such as hospital air, devices, and surfaces contaminated by infectious aerosols (Abou-ElWafa et al., 2021). One study conducted by Musa et al. compared the prevalence of SARS-CoV-2 infection in HCW in different hospital departments and found that those working in emergency medicine or gastroenterology have higher rates of infection compared to those in other departments including oncology and pediatrics. Indeed, HCWs in these settings are in frequent encounters with aerosol-generating procedures in patients with unknown SARS-CoV-2 status (Rana, 2020; Musa et al., 2021). Other studies in Egypt, on the other hand, have shown that patients’ cleaners/transporters and administrative employees are more affected by SARS-CoV-2 in terms of infection compared to other frontline workers (Kassem et al., 2020; Abdelmoniem et al., 2021; Musa et al., 2021). This finding could be attributed to the fact that in many cases, HCWs with more patient-near contact have greater awareness about infection control measures and the proper use of PPE (Musa et al., 2021). Most of the affected HCW in this country, presented with asymptomatic infection detected by serological testing or nasopharyngeal screening (Abdelmoniem et al., 2021; El-Sokkary et al., 2021; Mostafa et al., 2021). Among symptomatic HCW, the most common symptoms reported were fever, sore throat, headache, and myalgia (Kassem et al., 2020; Musa et al., 2021). In one study, 48.5% of those infected had moderate disease, 30.4% had mild disease and 21.1% had severe/critical disease. The rate of hospitalization was 35.8% with 3.9% of them being admitted to the ICU (El-Raey et al., 2021). Disease severity of COVID-19 was independently related to the associated chronic diseases in the affected HCW. No death was reported; this is except for the aforementioned study where the death rate was found to be 0.5%. The younger age of the participating HCW with less likely accompanied comorbidities could be the reason behind this observation (El-Raey et al., 2021). Healthcare facility and contact with a confirmed COVID-19 case during work were the most common sources of infection for HCWs (Kassem et al., 2020; Abou-ElWafa et al., 2021; El-Raey et al., 2021; Mostafa et al., 2021). Interestingly, many healthcare workers admitted that they do not know exactly where they were infected (Abou-ElWafa et al., 2021; El-Raey et al., 2021). This emphasizes on the role played by “asymptomatic silent spreaders of COVID-19″ in the transmission of SARS-CoV-2 from one person to another. HCW with unknown infectious source could have acquired the virus from patients at the hospital, work colleagues, relatives, friends, or household contacts (Abou-ElWafa et al., 2021).

In Iran, nurses, personnel of the emergency wards, and physicians constituted also the majority of the infected cases among HCW (Balou et al., 2021; Sabetian et al., 2021). In one study, it was found that HCW with rotational shifts were more likely to get infected compared to those with fixed shifts. This higher susceptibility could reflect different COVID-19 exposures among evening, afternoon, and morning shifts, in addition to the weakened immune system influenced by night shifts as reported by other studies (Loef et al., 2019; Lankarani et al., 2021). Other risk factors identified for the acquisition of SARS-CoV-2 among HCW in Iran include age, non-proper disposal of used PPE/infectious wastes, and anxiety about getting COVID-19 (Lankarani et al., 2021). The source of SARS-CoV-2 infection among Iranian HCW was mainly *via* occupational exposure (Goshayeshi et al., 2021; Sabetian et al., 2021). In fact, it is worth mentioning that occupational exposure is not restricted to contact with SARS-CoV-2 infected patients solely but also includes contaminated work environment and disregard of safety precautions in contaminated workspace (Sabetian et al., 2021). The COVID-19 disease among HCW in Iran is manifested mainly by an asymptomatic infection followed by a symptomatic one with atypical symptoms such as myalgia and cough and symptomatic with typical symptoms such as cough and fever (Armin et al., 2020; Goshayeshi et al., 2021; Mortezagholi et al., 2021; Sabetian et al., 2021). Twenty hospital admissions, six ICU admissions, seven mechanical ventilation requirement, and two deaths were reported among HCW of this country (Armin et al., 2020; Goshayeshi et al., 2021; Sabetian et al., 2021).

In the Kingdom of Saudi Arabia, two studies found that there is no statistical difference between different job categories and hospital departments in the acquisition of SARS-CoV-2 among HCW (Alshahrani et al., 2020; Alroqi et al., 2021). In another two studies, it was found that nurses were the most commonly affected (Al Bujayr et al., 2021; Barry et al., 2021). One plausible explanation for these findings is the standardization of the Saudi national guidelines for PPE that are adopted from the guidelines of the WHO (Alroqi et al., 2021). In addition, the healthcare workforce in KSA relies mainly on the nursing-related occupation that have more frequent contact and longer exposure time with patients (Barry et al., 2021). In this country, hospital and community settings played both the role of an infection source for HCW (Barry et al., 2020; Alshamrani et al., 2021). Healthcare-associated infections include exposure to a positive COVID-19 patient as well as exposure to other infected HCW (Al Bujayr et al., 2021). Community-acquired infections, on the other hand, include social gatherings, shared transportation, returning travelers, and family members (Barry et al., 2021). Indeed, in one study, it was found that the odds of testing seropositive for COVID-19 IgG are higher among those that have an infected family member and were not associated with ICU employment or involvement in the intubation and close contact with infected patients (Farsi et al., 2021). This emphasizes that infection control measures should be implemented, followed, and strictly monitored in and outside the clinical setting. Infected HCW in Saudi Arabia are mostly asymptomatic (Alshahrani et al., 2020; Barry et al., 2020). The most common symptoms reported in symptomatic patients include gastrointestinal ones (Alshahrani et al., 2020), cough, fever, body aches (Alroqi et al., 2021; Barry et al., 2021), headache, and dry throat (Alhabbab et al., 2021). Notably, in one study, the majority of infected healthcare workers were symptomatic with 198 reported deaths. Death was mostly related to the age groups of 46 years and above (Al Bujayr et al., 2021). Interestingly, Alshamrani et al. conducted a study where they compared the clinical outcomes of infected HCW and non-HCW. They found that the higher risk of ICU admission and hospitalization increases three and two times, respectively in non-HCW compared to HCW (Alshamrani et al., 2021). This could be attributed to the lower comorbidities, younger age, and better awareness of the healthcare personnel (Alshamrani et al., 2021; Gholami et al., 2021).

In Qatar, the least affected groups of healthcare workers were the clinical employees compared to outsourced and non-clinical staff (Al-Kuwari et al., 2021; Alishaq et al., 2021a). Al-Kuwari et al. found no significant difference of SARS-CoV-2 rates among employees who worked in a COVID-19 healthcare facility versus other ones (Al-Kuwari et al., 2021). Al-Ajmi et al., on the other hand, found that midwives and nurses have the highest rate of SARS-CoV-2 infection followed by non-clinical support service staff, administrative employees, allied healthcare professionals, physicians, and others (Alajmi et al., 2020). In that same report, the majority of the infected HCW reported working in a non-COVID-19 facility (Alajmi et al., 2020). Regarding risk factors, one study found that HCW aged <45 years have a higher infection rate (Al-Kuwari et al., 2021); whereas in another, it was found that, compared to age groups <30, older ages were significantly associated with a lower risk of infection (Alishaq et al., 2021a). HCW are a heterogeneous group of population and the risk of infection may differ based on several variables, including exposure to an unrecognized infection among patients (Alajmi et al., 2020), level of community exposure, and adherence to infection control measures. As for the disease presentation, only one study conducted found that two-thirds of the included HCW are symptomatic with fever, cough and sore throat being the most common symptoms reported. The hospitalization rate was 15.4%, and of the survey respondents in this study, nine, four, and two required supplemental oxygen were ICU admitted and required mechanical ventilation, respectively. Furthermore, zero death was reported (Alajmi et al., 2020).

In the Sultanate of Oman, most of the infected HCWs were nurses followed by doctors (Al Abri et al., 2021; Al Maskari et al., 2021; Al-Siyabi et al., 2021). Indeed, Maskari et al. found that being a doctor or a nurse is significantly associated with the acquisition of COVID-19 inside the hospital (Al Maskari et al., 2021). One study reported that nurses followed by administrative and supporting services staff are the most affected groups compared to medical doctors (Al-Maani et al., 2021). Nurses are frontline workers, and specific procedures such as aerosol-generating ones, electrocardiography, and others involved in direct patient contact (incubation assistance, suctioning, and manipulation of oxygen face masks) increase their risk of SARS-CoV-2 infection (Loeb et al., 2004; Chou et al., 2020; Al Abri et al., 2021). Moreover, on the contrary, Al-Naamani et al. found that supportive staff and not nurses or physicians are the mostly affected by the virus (Al-Naamani et al., 2021). Throughout the studies, we found that older age (above 50 years; Al-Naamani et al., 2021), availability of N95 masks, reuse of personal protective equipment, inadequate sanitization and disinfection of hospital surfaces and medical equipment, lack of education and training of cleaners, small size of the facility restricting social distancing, poor ventilation, and patient overcrowding are all risk factors that increases the chance of contracting the COVID-19 disease inside the healthcare facility (Al Abri et al., 2021). The sources of SARS-CoV-2 among healthcare workers in Oman appear to be both hospital and community acquired (Al Abri et al., 2021). In one study, community acquisition was the most common (Al Maskari et al., 2021) while in another one, hospital acquisition was the most common (Al-Siyabi et al., 2021). Interestingly, in both studies, in roughly equal percentages of HCW, the source of infection could not be determined. Inside the hospital, noncompliance with social distancing and wearing masks in eating times, in addition to patients’ exposure, are all routes of transmission of COVID-19 among HCW (Al Maskari et al., 2021; Al-Siyabi et al., 2021). Regarding the clinical presentation of COVID-19 among HCW in Oman, it was found that the majority presented with a mild illness with five hospital admissions, one ICU admission, and zero death reported. The most common symptoms were fever, headache, cough, and sore throat (Al Lawati et al., 2021; Al Maskari et al., 2021; Al-Siyabi et al., 2021).

In the Levant (Lebanon, Syria, Jordan, and Palestine) and the rest of the Gulf countries, scarce studies exist on the clinical impact of COVID-19 on HCW. In the UAE, it was found that support staff including housekeeping, facility and catering staff, porters, and security guards are more likely to get infected with SARS-CoV-2 compared to the non-support ones. Non-support HCW include physicians, nurses, allied health professionals, and administrative staff (Park et al., 2021).

In Kuwait, Al-Youha et al. reported that the likelihood of contracting the SARS-CoV-2 infection is significantly associated with working as a nurse and wearing gloves (Al Youha et al., 2021). A possible explanation for this latter finding is that, due to poor hand hygiene practices, extended gloves use could result in greater contamination. Furthermore, due to possible PPE shortages, HCWs are less likely to change their gloves when dealing with different patients and procedures (Neuwirth et al., 2020). Contact with COVID-19 patients was reported in more than two-thirds of the included HCW in the aforementioned study in Kuwait (Al Youha et al., 2021).

In Yemen, one study reported 17-infected HCWs after contact with two confirmed COVID-19 patients inside the hospital; only two of the infected HCW needed hospitalization (Al-Sakkaf et al., 2021). In Syria, a report from the North West region reported that due to the protracted conflict in the area, HCWs face several challenges related to inadequate PPE, insufficient resources, poor infection and prevention control measures/practices, and severe under-staffing. The first confirmed case in this area was a doctor working at a border-located hospital. Thereafter, a cluster was noted among hospital staff, in addition to several community ones. Five physicians and one nurse have died (Almhawish et al., 2021). In Israel, it was reported that one COVID-19-positive ophthalmologist transmitted the infection to a visiting patient that was not wearing a mask (Saban et al., 2020). On the other hand, Temkin et al. reported the asymptomatic infection of a nurse working in a COVID-19 unit (Temkin and Healthcare Worker COVID-19 Surveillance Working Group, 2021).

Taking it overall, in the middle eastern countries, notably in Egypt, Iran, and KSA where many studies were conducted, a substantial proportion of healthcare workers infected with SARS-CoV-2 were asymptomatic. Worldwide speaking, the percentage of asymptomatic infections among HCW varies from 0% to 8.2% in the United States (Campbell et al., 2020; Jameson et al., 2020; Stock et al., 2020), 2.2% in France (Guery et al., 2020), 2.7% in Italy (Cavicchiolo et al., 2020), 5% in Spain (Moncunill et al., 2021), 7.5% in the United Kingdom (Hellewell et al., 2021), and 11.1% in Indonesia (Hidayat et al., 2020). The varying numbers could be explained by regional differences in the incidence of COVID-19, together with the difference in the baseline features of the pandemic in each country. Indeed, it has been suggested that among asymptomatic HCW, higher rates of positivity can be expected when the incidence of the virus in the general population increases; this is possibly due to a higher probability of exposure to a confirmed infected case outside the hospital (Jabs et al., 2022). The main concern about asymptomatic carriers, as mentioned earlier, is the unrecognizable transmission of COVID-19 from the asymptomatic patient to other high-risk subjects in the clinical setting as well as in the community. Throughout the literature, it remains under debate the extent to which asymptomatic infections contribute to disease transmission and continuous spread. In fact, what is pretty sure is that asymptomatic carriers are infectious. The level of infectivity is however complex and linked to several factors including viral load, duration of viral shedding, age, comorbidities, and immune responses (Wang et al., 2022). In a systematic review conducted by Byambasuren et al., it was found that asymptomatic infection with SARS-CoV-2 is unlikely to be the main driver of community transmission (Byambasuren et al., 2020). On the other hand, Yang et al. found that from October 2020 to February 2021, out of five COVID-19 outbreaks in China, four were caused by asymptomatic infections (Yang et al., 2021). In the Middle East, the impact of asymptomatic carriage on the spread of SARS-CoV-2 cannot be deduced since no studies explored the viral load, duration of viral shedding nor the immune responses in asymptomatic healthcare workers or other patients’ categories.

### Breakthrough infections

It is worth mentioning that all the studies addressing COVID-19 in HCW (as well as other in other patients’ categories), in the middle eastern region, were conducted before the vaccination campaigns have started, and thus the effect of vaccines could have not impacted the findings. Frontline workers were the first to take the COVID-19 vaccine in all middle eastern countries. Vaccination campaigns started in December 2020 in KSA (Assiri et al., 2021), UAE (Sahib, 2020), Bahrain (Gazette, 2020), Kuwait (Al-Ayyadhi et al., 2021), Qatar (Alishaq et al., 2021b), Sultanate of Oman (Al Rawahi et al., 2022), Jordan (Al-Shaikh et al., 2021), and Israel (Rosen et al., 2021); January 2021 in Egypt (ElSharkawy, 2021); February 2021 in Iran (Heidari and Jafari, 2021), Palestine (Mukhtar, 2021), and Lebanon (Mumtaz et al., 2021); March 2021 in Yemen (Reuters, 2021); and May 2021 in Syria (WHO in Syria, 2021) and Iraq (Abdulah, 2021).

Breakthrough infections defined as the detection of SARS-CoV-2 RNA or antigen in a respiratory specimen post ≥14 days after the receipt of all recommended doses of the COVID-19 vaccine (Center for Disease Control and Prevention, 2021) were described in HCW from KSA, Qatar, and Israel. For instance, in the Kingdom of Saudi Arabia, in one study, 20 HCWs who all received the first dose and four only received the second dose of the COVID-19 vaccine were infected. The majority were physicians and nurses. The infection was asymptomatic in seven, and the rest exhibited mild-to-moderate disease. The most common symptoms were fever, cough, headache, malaise, and sore throat. In the majority of the included HCW in this study, the source of infection could not be recognized (Alshamrani et al., 2022). In Qatar, Alishaq et al. reported 164 breakthrough infections among HCWs. History of contact with a confirmed case was independently associated with a higher risk of infection. Interestingly, the presence of comorbidities was not associated with a higher risk of breakthrough infection. Moreover, almost all job families, i.e., clinical and non-clinical, support staff had a higher risk of breakthrough infection compared to nurses (Alishaq et al., 2021b). In Israel, one study found that symptomatic breakthrough infections occurred in eight HCW fully vaccinated compared to 38 unvaccinated ones. Asymptomatic infection, on the other hand, occurred in 19 and 17 fully vaccinated and unvaccinated HCW, respectively (Angel et al., 2021). Oster et al., reported one hospitalization in HCW who got infected with SARS-CoV-2 after at least 2 weeks of receiving the 2nd dose of the COVID-19 vaccine; this is versus two hospitalizations in the unvaccinated group, with no death recorded in both of them. As for the source of infection, exposure to a positive household member was more common in vaccinated subjects vs. unvaccinated ones, with a significant statistical difference (Oster et al., 2021). Similarly, in another study, the main source of breakthrough infection in HCW was also community-related. In this latter, the most common symptoms were influenza-like illness including fever, chills, cough, headache, and sore throat (Amit et al., 2021). Moreover, in a study conducted by Bergwerk et al., on breakthrough infections with follow-up, up to 6 weeks, “long COVID-19 symptoms” were reported; these included persistent cough, fatigue, weakness, dyspnea, or myalgia, in addition to prolonged loss of smell. Similar to the study in KSA, HCW experienced mainly mild disease followed by an asymptomatic one (Bergwerk et al., 2021). Interestingly, in this study among 33 isolates of breakthrough infections tested for a variant of concern, 28 were found to be the B.1.1.7 (alpha) variant. According to the authors, at the time of the investigation, this variant was the most commonly spread in Israel and accounted for up to 94.5% of the SARS-CoV-2 isolates (Bergwerk et al., 2021; Haas et al., 2021; Kustin et al., 2021).

## High-risk population

As mentioned in the introduction, COVID-19 patients with underlying medical conditions, especially those with an immunocompromised system, either due to an underlying disease or being on immunosuppressive medications, are at a higher risk of developing severe complications and poor prognosis of the SARS-CoV-2 infection (Kim et al., 2021). In diabetic patients, for example, several studies revealed their higher vulnerability to some infectious diseases. This is could be in particular due to their high levels of blood glucose, on which the virus may thrive (Robert et al., 2021), and can damage the immune system defense mechanisms, triggering diabetes-related problems such as nerve damage. Furthermore, the impaired blood flow increases the patient’ susceptibility to infection (Jeong et al., 2020; Al Hayek et al., 2020b). As for cancer patients, poor functional status could increase their risk of poor outcomes for COVID-19 (Blimark et al., 2015; Mousavi et al., 2021). Other categories such as hemodialysis (HD) patients and solid organ transplant patients can also experience poor prognosis of COVID-19. This is owing to their weak immune system and their comorbidity/multimorbidity (Cheng et al., 2020; Kenarkoohi et al., 2022). In this section, we will review the current knowledge about the prognosis and clinical outcomes of SARS-CoV-2 infection in high-risk categories in the Middle East.

### Diabetes

In the Kingdom of Saudi Arabia, the most common symptoms of SARS-CoV-2 infection in type 1 diabetic patients (T1DM) observed in one study were nausea, vomiting, followed by fever, cough, sore throat, abdominal pain, and dyspnea (Al Hayek et al., 2020a). Type 1 Diabetic patients with COVID-19 were found to have a greater comorbidity percentage and lower mean of Vitamin D levels, in addition to higher ferritin and average D dimer levels. This is compared to non-infected diabetic patients and control groups (Ahmed et al., 2021). Diabetes biomarker levels including fasting blood glucose (FBG) and HbA1c were also higher in infected patients with T1DM. This result is in accordance with the findings of a study conducted in China that reported higher levels of FBG in diabetic patients infected with the SARS-CoV-2 virus (Cai et al., 2020). Indeed, studies have shown that FBG and Hb1Ac are associated with the progression of the COVID-19 illness in diabetic subjects (Wang et al., 2020; Ling et al., 2021). The rate of hospitalization of T1DM infected with SARS-CoV-2 ranges from 21.9% to 48% (Al Hayek et al., 2020a). The most common reason for hospitalization was diabetic ketoacidosis, followed by hyperglycemia, bacterial pneumonia, COVID-19 pneumonia, fever, and sore throat (Al Hayek et al., 2020a). Similarly, high incidence of ketoacidosis was also observed in hospitalized patients because of COVID-19, in Germany and in India (Kamrath et al., 2020; Reddy et al., 2020). In type 2 diabetic COVID-19 patients, ages between 70 and 79 and above 80 years are more likely to be hospitalized compared to those <40 years old. Furthermore, those with higher HbA1c levels, presence of comorbidities such as chronic kidney disease (CKD), chronic pulmonary disease, cerebrovascular disease, cardiovascular disease (CVD), hypertension, and insulin-treated ones are more likely to get hospitalized (Al Hayek et al., 2020b). Alguwaihes et al. found that SARS-CoV-2 infected DM patients have significantly lower survival time and higher death rates compared to non-infected patients with diabetes. However, after adjustment for confounders like age, sex, body mass index (BMI), and pre-existing medical conditions; diabetes was not associated with mortality (Alguwaihes et al., 2020). The result is in line with a study conducted in the United States that found no association between diabetes, and risk of ICU admission, mechanical ventilation, and mortality (Suleyman et al., 2020). In contrast, another study conducted in the United Kingdom found that one-third of COVID-19 patients who died in hospitals had diabetes (Hillson, 2020). The lack of association between diabetes and poor clinical outcomes does not eliminate the fact that diabetic patients have a major risk of poor prognosis of COVID-19; however, this suggests that the increased risk of poor outcome is due to the cumulative effect of the presence of diabetes mellitus together with other chronic conditions (Alguwaihes et al., 2020; Apicella et al., 2020; Maddaloni et al., 2020).

In Iran, one study has shown that the most common symptoms of COVID-19 in hospitalized patients with diabetes, defined as fasting plasma glucose ≥126 mg/dl, were fever, dry cough, and dyspnea (Akbariqomi et al., 2020). At admission, laboratory findings indicated increased white blood cells (WBCs) counts and neutrophils and decreased lymphocyte count compared to infected patients without diabetes. This shows that infected patients with DM experienced a severer viral infection. Furthermore, blood urea nitrogen levels were also increased in COVID-19 DM patients, suggesting the occurrence of kidney damage (Akbariqomi et al., 2020). The mortality rate among DM patients infected with SARS-CoV-2 was found to be 22% in one study. The clinical outcome did not however differ significantly between patients with well-controlled and poorly-controlled DM (Raoufi et al., 2020). In infected patients, the mortality rate was higher in those with DM compared to those without (Akbariqomi et al., 2020; Pazoki et al., 2021). Interestingly, unlike what has been reported in KSA, a study conducted by Akbariqomi et al., found that compared to COVID-19 patients lacking DM and any other comorbidity, those that are infected and have DM only, still experience more complications and deaths (Akbariqomi et al., 2020). In Iran, it was found that advanced age, addiction, high levels of blood urea nitrogen, and alkaline phosphatase are associated significantly with increased odds of death in DM COVID-19 patients (Borzouei et al., 2021).

In the UAE, one study has shown that among hospitalized COVID-19 patients with diabetes (including known and newly diagnosed diabetes), 80.8% had moderate-to-severe disease, and 19.4% had mild disease without pneumonia. In terms of the severity of the COVID-19 illness, no statistical difference was found between those with pre-existing diabetes and those with a newly diagnosed one. Only, the requirement for mechanical ventilation, and mortality rate were significantly higher in patients with newly diagnosed diabetes compared to those with a pre-existing one (Hafidh et al., 2020). Bhatti et al. conducted a study and found that at admission, laboratory findings of infected diabetic patients (87.4% being type 2 diabetes) indicated higher levels of fibrinogen, D-dimer, ferritin, and C-reactive protein (CRP) in those admitted to the ICU. These biomarkers can help in assessing in advance the more likely clinical course of the disease, and the patient subsequent need for intensive care (Bhatti et al., 2020). The length of hospital stay, in this same study, was longer in ICU admitted patients. The mortality rate was 4.9% among those who were admitted to the ICU. Similar to the study conducted in the UAE, the most common comorbidities detected in these patients were hypertension, ischemic heart disease, and dyslipidemia (Bhatti et al., 2020).

In Qatar, one study compared the clinical outcomes of hospitalized COVID-19 patients among type 2 diabetics and non-diabetics. It was found that diabetic patients had a higher prevalence of chronic kidney disease, hypertension, congestive heart failure, and cardiac dysfunction. They had also significantly higher percentages of pneumonia, severe pneumonia, and acute respiratory distress syndrome (ARDS). Hematological speaking, diabetic patients had significantly higher CRP levels, absolute neutrophil counts, but lower counts of lymphocytes and eosinophils compared to the non-diabetics. CRP was correlated significantly with the duration of stay in the intensive care unit, as well as to the duration of oxygen supplementation (Soliman et al., 2020b; Table 1). In Kuwait, diabetes was found to be associated with ICU admission in hospitalized COVID-19 patients after adjustment of confounding factors (Al-Sabah et al., 2020). In this study, diabetes was defined as a fasting blood glucose level of ≥126 mg/dl (Al-Sabah et al., 2020). In Egypt, Emara et al. reported three cases of type 2 diabetic patients who presented at the outpatient department with diabetic ketoacidosis. A few days later, these patients developed fever and hypoxemia and turned out to be SARS-CoV-2 positive. All three cases were hypertensive and dyslipidemics, and one case passed away due to severe hypoxemia (Emara et al., 2020). In Palestine, it was found that COVID-19 patients with diabetes were more likely to be hospitalized in comparison to those without diabetes (Hamdan et al., 2021). In Israel, one study found that in SARS-CoV-2 diabetic patients, HemoglobinA1c ≥9% significantly increased the risk of hospitalization (Merzon et al., 2021).

**Table 1:** Comparison of COVID-19 high-risk groups vs. healthy subjects in the Middle East.

**Category**	**Country**	**Study group**	**Control group**	**Clinical outcome**	**Laboratory outcome**	**Reference**
HCW	KSA	HCWP	Non-HCWP	More common symptomatic disease		Alshamrani et al., 2021
				Less frequent hospitalization, ICU admission, and case fatality		
				Significantly lower mortality per 100,000 population		
Diabetes	Iran	COVID-19 w. DM	COVID-19 w/o DM	Significantly higher % of in-hospital deaths		Pazoki et al., 2021
				Significantly higher % of 1-month deaths		
				Significantly higher % of 7-month all-cause deaths		
		Hospitalized COVID-19	Hospitalized COVID-19	More severe disease	Significant decrease of lymphocyte	Akbariqomi et al., 2020
		w. DM	w/o DM	Higher mortality rate	Significant increase of WBC and neutrophils	
					Significant increase of BUN and kidney damage indicator	
	KSA	Hospitalized COVID-19	Hospitalized COVID-19	Higher death rate		Alguwaihes et al., 2020
		w. DM	w/o DM	Lower survival time		
		T1DM w. COVID-19	T1DM w/o COVID-19	Significantly higher of % of comorbidities	Higher level of diabetes biomarker	Ahmed et al., 2021
		T1DM w. COVID-19	T1DM w/o COVID-19		Significantly higher mean ferritin level	
			and w/o T1DM/COVID-19		Significantly lower level of Vitamin D	
	Qatar	T2DM w. COVID-19	Non-diabetic COVID-19	Higher Prevalence of comorbidities	Significantly higher CRP level and absolute neutrophilic count	Soliman et al., 2020b
				Significantly higher % of pneumonia, severe pneumonia, ARDS	Significantly lower counts of lymphocytes and eosinophils	
				Significantly longer duration of hospitalization, ICU stay		
				mechanical ventilation and oxygen therapy		
Cancer	Iran	COVID-19 w. malignancy	COVID-19 w/o malignancy	Lower rates of comorbidities		Shahidsales et al., 2021
				Significantly higher mortality rate		
				Increased risk of death		
				Increased risk of mechanical ventilation		
	Oman	COVID-19 w. malignancy	National population	Significantly higher fatal outcome	Lower frequency of high WBC count, ferritin,	Al Bahrani et al., 2021
				Higher frequency of Septicemia	hypocalcemia, transaminases, and renal impairment	
					More common elevated LDH and high troponin levels	
	UAE	COVID-19 w. malignancy	Malignancy w/o COVID-19	More likely to be hospitalized		Al-Shamsi et al., 2021
				More frequent numerical death^***^		
Other high-risk	Iran	COVID-19 HD	COVID-19 non-HD	Higher mortality rate^***^	Higher absolute counts of WBC	Tayebi Khosroshahi et al., 2021
					Higher absolute counts of polymorphonuclears	
Pregnant Women	Iran	Pregnant w. COVID-19	Pregnant w/o COVID-19	Significantly higher rate of cesarean section		Abedzadeh-Kalahroudi et al., 2021a
				Poorer maternal outcomes		
				Higher rate of pre-eclampsia		
				Higher rate of preterm labor		
				Higher rate of fetal distress		
		Pregnant w. COVID-19	Non-pregnant w. COVID-19	Lower frequency of severe disease	Higher neutrophil count	Vizheh et al., 2021
				Higher prevalence of comorbidities	Lower lymphocyte count	
				Shorter mean duration of hospitalization	Lower levels of ESR	
				Higher % of ICU admission^***^	Lower levels of CRP	
				Lower Frequency of ARDS		
		Pregnant COVID-19	Pregnant COVID-19	Higher odds of preterm labor		Ghelichkhani et al., 2021
		w. underlying diseases	w/o underlying diseases	Higher odds of preeclampsia		
				Higher odds of eclampsia		
				Higher C-section rates		
		Pregnant w. COVID-19	Pregnant w/o COVID-19	Significantly higher risk of ICU admission, C-section		Alipour et al., 2021
				Significantly higher risk of preterm birth, fetal distress and NICU admission		
		Pregnant w. COVID-19	Pregnant w/o COVID-19	Significantly higher rate of ICU admission		Pirjani et al., 2020
				Significant difference in terms of delivery type		
		Pregnant w. COVID-19	Familial/households w. COVID-19	More severe maternal outcomes		Hantoushzadeh et al., 2020
	Israel	Pregnant w. COVID-19	Non-pregnant w. COVID-19	Significantly lower pCO2	Reduced relative lymphocyte count	Mohr-Sasson et al., 2020
				Significantly elevated base excess		
				Less common hospitalization		
				Shorter duration of hospitalization		
		Pregnant w. COVID-19	Non-pregnant w. COVID-19	Less likely to have chronic diseases	Higher levels of WBC count	Barber et al., 2021
				Less likely to be hospitalized	Higher levels of absolute neutrophil count	
		Pregnant women w. COVID-19	Pregnant w/o COVID-19	2.1 aOR for composite neonatal adverse outcome		Harel et al., 2021
				1.6 aOR for overall composite adverse outcome		
	Egypt	Pregnant w. COVID-19	Non-pregnant w. COVID-19	Higher proportion of cases w. underlying diseases		BahaaEldin et al., 2021
				More severe symptoms		
				Less likely to be asymptomatic		
				More likely to be hospital admitted		
				More likely to be ICU admitted		
				More likely need of invasive mechanical ventilator		
	UAE	Pregnant w. COVID-19	Non-pregnant w. COVID-19	More ICU admission	Higher CRP and D-dimer levels	Hazari et al., 2021
				More complications of COVID-19 infection	Significantly higher mean of WBC count	

Note that all descriptions are put as the study vs the control group. w., with; w/o, without; ICU, intensive care unit; ESR, erythrocyte sedimentation rate; CRP, C reactive protein; WBC, white blood cells; BUN, blood urea nitrogen; NICU, neonatal intensive care unit; %, percentage; HCW, healthcare worker; HCWP, healthcare worker personnel; T1DM, type 1 diabetes mellitus; DM, diabetes mellitus; HD, hemodialysis; ARDS, acute respiratory distress syndrome. ***p>0.05.

The poorer outcome observed in diabetic patients infected with SARS-CoV-2 in the majority of the middle eastern countries is supported by findings from China (Chen N et al., 2020), where it was found that the rates of mortality are higher in diabetic patients compared to the general population with COVID-19. Another study from the United States reported a longer length of hospital stay as well as a higher mortality in COVID-19 patients with diabetes versus those without (Bode et al., 2020). Furthermore, a nationwide study conducted in Sweden indicated that increased risk of hospitalization and ICU admission, in addition to death, was associated independently with type 2 diabetes (Rawshani et al., 2021).

### Cancer

In Iran, several studies assessed the clinical outcome of COVID-19 in cancer patients (Mousavi et al., 2021; Rakhsha et al., 2021; Shahidsales et al., 2021). In one, it was found that compared to hospitalized patients without malignancy, malignant patients have an increased risk of death and mechanical ventilation; although these latter have less rates of comorbidities (Shahidsales et al., 2021). No factor such as the type of malignancy neither stage of the disease or recent oncological treatment could predict death in infected cancer patients. Similarly, in another study, the type of cancer could not predict mortality in hospitalized COVID-19 patients. The mortality rate was however higher in those with a history of lung cancer with lung metastasis due to non-pulmonary cancer and in those who received cytotoxic chemotherapy within the last 14 days (compared to those who were on cancer treatment more than 2 weeks prior to admission with COVID-19; Mousavi et al., 2021). This finding is in agreement with a study conducted by Dai et al., who found that the mortality rate among COVID-19 patients with lung cancer is higher compared to other cancer types and that the cancer stage directly correlates with mortality (Dai et al., 2020). In contrast, another study in the United Kingdom argued that lung cancer patients are not specifically subject to a higher mortality when infected (Lee et al., 2020). More studies with a larger sample size are needed to confirm whether lung cancer patients have a higher risk of death from COVID-19. As for COVID-19 prognosis, in patients with malignancies in Iran, dyspnea, fever, dry cough, and gastrointestinal symptoms were the most commonly reported. During hospitalization, most of the patients received oxygen therapy, around half received invasive ventilation and only 10% received a non-invasive one. Admission to an ICU was reported in around half of the hospitalized patients with cancer. The most common complications were acute respiratory distress syndrome, sepsis, septic shock, and pulmonary thromboembolism. In this same aforementioned study, death was reported in almost half of the patients (Mousavi et al., 2021). This rate was similar to another report from Iran where 4 out of 7 patients with cancer died due to COVID-19 (Aznab, 2020). Asymptomatic infection detected by serological testing was reported in one study in Iran, conducted by Arab et al. (2021).

In the United Arab Emirates, the majority of studies were conducted in asymptomatic cancer patients. In one, none of the patients developed any COVID-19-related symptoms (Iskanderian et al., 2020), whereas in the other two studies, symptoms subsequently appeared (Al-Shamsi et al., 2020, 2021). The SARS-CoV-2 infection was mild in the majority of the reported cases with few requiring hospital and ICU admission. Only five deaths were reported (Al-Shamsi et al., 2020, 2021; Nawazani et al., 2020). These findings emphasize the need of a scheduled screening for cancer patients regardless of their symptom presentation as these can act as hidden reservoirs for nosocomial transmission of SARS-CoV-2 among high-risk population and critically ill patients.

In Qatar, only two cases of SARS-CoV-2 infection among cancer patients were reported. In one, the patient presented with COVID-19 symptoms in addition to absolute lymphocytosis that with further workup found out to be chronic lymphocytic leukemia (CLL). The patient had moderate disease that did not progress to a severe form as expected. This is according to the authors might be due to the defective immune response in CLL patients that prevented cytokine storm and the subsequent multi-organ involvement (Ali et al., 2020). The second case reported in Qatar was in a patient with hair cell leukemia, who presented with severe respiratory symptoms but recovered later (Kohla et al., 2020). In Oman, it was found that the fatal outcome was higher in cancer patients with COVID-19 compared to those without. Cardiac diseases, hypertension and diabetes were likely risk factors for SARS-CoV-2 infection in patients with malignancy (Al Bahrani et al., 2021). In Israel, one study conducted on patients with hematological malignancies and infected with SARS-CoV-2 found that age > 70 years, hospitalization, severe/critical disease, hypertension with severe/critical disease, and active hemato-oncological treatment with hospitalization were associated with mortality. Around one-third of the patients developed severe/critical respiratory infection. Death was reported in one-fifth of the infected patients with cancer (Levy et al., 2021). In a recent meta-analysis conducted by Di Felice et al., it was found that infected patients with cancer have a twofold higher risk of experiencing a severe form of the COVID-19 illness as well as ICU admission compared to non-cancer patients. The association of SARS-CoV-2 infection with mortality in patients with malignancies was stronger in studies from Asia than from those of North America and Europe (Di Felice et al., 2022). In our region, studies on COVID-19 patients with cancer are scarce and include only a small number of subjects. More studies with a larger sample size are needed to draw a definitive conclusion about the prognosis of COVID-19 in this group of patients. Regarding antiviral treatment, Levy et al. found that remdesivir treatment was associated with decreased mortality in hemato-oncological patients infected with SARS-CoV-2 (Levy et al., 2021). No other studies in the Middle East explored the relationship between the administered therapeutic regimen and the clinical outcome of COVID-19.

### Other high-risk categories

In Iran, one study has shown that in COVID-19 patients, chronic kidney disease was associated with an increased risk of mortality (Mirjalili et al., 2021). On the other hand, in patients undergoing hemodialysis, hereditary kidney failure and blood group A were associated with higher COVID-19 morbidity. Compared to SARS-CoV-2-infected patients without hemodialysis requirement, it was shown that those “who require hemodialysis”, have only higher absolute counts of WBC, and polymorphonuclears. Clinical biomarkers for COVID-19 in hemodialysis patients should be therefore more explored to help better management of the disease in this group of people. Moreover, a study conducted also in hemodialysis patients infected with SARS-CoV-2 reported that the most common symptoms were dyspnea and cough. The majority of the patients were hospitalized, 12.9% needed intensive care and 16.1% died (Kenarkoohi et al., 2022).

In kidney transplant recipients, in Iran, the most common symptoms observed in COVID-19 patients were myalgia, cough, fever followed by headache, shortness of breath, and sore throat. Acute kidney injury (AKI) was the most common complication observed, and death occurred in half of the patients due to the SARS-CoV-2 infection (Rahimzadeh et al., 2021). The mortality rate was higher in ICU patients versus those who did not require intensive care. Pre-transplantation diabetes was the only factor associated with an increased risk death in COVID-19 patients (Rahimzadeh et al., 2021). In another study, fever and cough were also the most common symptoms observed in COVID-19 kidney transplant patients. Diabetes mellitus and hypertension were considered underlying diseases in this group (Molaei et al., 2021). As for the source of infection, around one-fourth acquired the virus from a family member (Rahimzadeh et al., 2021). This highlights that kidney transplant recipients should keep their own distance even within their families.

In the Kingdom of Saudi Arabia, one study conducted on COVID-19 patients with end-stage kidney disease showed that the mortality risk increases significantly with age and concurrent cardiovascular diseases. High D-dimer levels, neutrophilia, lymphocytopenia, and high blood urea nitrogen levels were also significant risk factors for death in this population. The most common complications resulting in death were septic shock, respiratory failure, and respiratory distress syndrome (Hakami et al., 2021). As for the symptoms, the most commonly reported were fever, shortness of breath, and cough. The majority of the patients were admitted to the hospital general ward with 17% only requiring ICU and intubation (Hakami et al., 2021). Al Maghrabi et al. reported four cases of fully vaccinated solid organ transplants (three renal and one liver), infected with SARS-CoV-2. All patients were hospitalized and admitted to the ICU. Two were infected with the alpha variant and died (one renal and one liver transplants) due to sepsis/septic shock and sepsis/septic shock with cytomegalovirus reactivation, respectively. The other two patients recovered with one being infected with the Beta variant and the other with the Delta variant (Almaghrabi et al., 2022). In Israel, one study reported six hemodialysis patients who got infected with SARS-CoV-2 after >7 days of taking the second dose of the mRNA BNT162b2 vaccine. Three patients experienced moderate disease, two mild, and one had severe illness. In two patients, the virus variant was determined and found to be B.1.1.7 – UK (L5F[S]; Yanay et al., 2021). No other studies reporting breakthrough infections in high-risk patients were conducted in the middle eastern region.

## Pregnant women and neonates

In the literature, studies have shown that the risk of SARS-CoV-2 acquisition in pregnant women is the same compared to the general population (Docherty et al., 2020). However due to the immunological and physiological changes that occur normally during pregnancy; this category might be at increased risk of severe illness. In fact, the maternal immune system faces challenges in tolerating the fetus, and at the same time defending the body from microbial infections (Hazari et al., 2021). Physiological changes, on the other hand, include cardiopulmonary ones such as respiratory tract edema, diaphragm elevation, and increased consumption of oxygen (Schwartz and Graham, 2020). All of these changes make pregnant women a vulnerable population. This is supported by the fact that previous coronaviruses like the severe acute respiratory syndrome (SARS) and the Middle East respiratory syndrome (MERS) were associated with increased maternal mortality and adverse outcomes in pregnancy and delivery (Samadi et al., 2021).

In Iran, the majority of pregnant women were infected during their third trimester (Abedzadeh-Kalahroudi et al., 2021b; Vaezi et al., 2021; Vizheh et al., 2021). This is in accordance with other studies in the Middle East as well as in other worldwide countries such as the United States (Hirshberg et al., 2020) and China (Chen H et al., 2020). In Iranian pregnant women, the most common reported symptoms of COVID-19 were fever, cough, and dyspnea (Kazemi Aski et al., 2020, 2021; Motlagh et al., 2020; Pirjani et al., 2020; Sattari et al., 2020; Akbarian-Rad et al., 2021; Alipour et al., 2021; Samadi et al., 2021; Vaezi et al., 2021; Vizheh et al., 2021; Abedzadeh-Kalahroudi et al., 2021b). Lymphopenia and elevated C-reactive protein levels (Pirjani et al., 2020; Akbarian-Rad et al., 2021; Vaezi et al., 2021; Abedzadeh-Kalahroudi et al., 2021a,b), in addition to Elevated lactate dehydrogenase (LDH; Kazemi Aski et al., 2020; Abedzadeh-Kalahroudi et al., 2021a), were the most common laboratory findings observed. The SARS-CoV-2 infection in this population presented mainly as a mild-to-moderate disease (Kazemi Aski et al., 2020; Vizheh et al., 2021). Samadi et al. found that the presence of underlying medical conditions is significantly associated with disease severity of COVID-19. In addition, infected pregnant women are more likely to develop a critical stage of the disease with increasing gestational and maternal age when having underlying diseases (Samadi et al., 2021). This in accordance with other studies conducted in the United States and the United Kingdom where it was found that hospitalized women with COVID-19 and have comorbidities, are more prone to severe illness (Knight et al., 2020; Zambrano et al., 2020). Although the majority of the pregnant women with COVID-19 were hospitalized, a minority of ICU admissions and maternal death were observed (Pirjani et al., 2020; Sattari et al., 2020; Alipour et al., 2021; Abedzadeh-Kalahroudi et al., 2021a). The most common mode of delivery of infected pregnant women was a cesarean section, performed mostly under obstetric indication (Pirjani et al., 2020; Alipour et al., 2021; Vaezi et al., 2021; Vizheh et al., 2021; Abedzadeh-Kalahroudi et al., 2021a), rather than due to complications of COVID-19 (Abedzadeh-Kalahroudi et al., 2021a). In one study only, it was found that the severity of the coronavirus disease was the only factor effective in increasing the rate of cesarean delivery (Samadi et al., 2021). In another study, COVID-19 in pregnancy was associated significantly with a higher risk of cesarean section (Alipour et al., 2021). Compared to pregnant women without COVID-19, those infected have lower gestational age (Pirjani et al., 2020; Alipour et al., 2021), and higher rate of ICU admission (Pirjani et al., 2020). Furthermore, it was reported that these latter have poorer maternal outcomes and higher rates of pre-eclampsia, preterm labor, and fetal distress (Alipour et al., 2021; Abedzadeh-Kalahroudi et al., 2021a). On the other hand, compared to non-pregnant infected women, pregnant women with COVID-19 have a lower frequency of severe disease, acute respiratory distress syndrome, and shorter mean duration of hospitalization (Vizheh et al., 2021; Table 1). In their study, Ghelichkhani et al. found that compared to infected pregnant women without underlying diseases, preterm labor, preeclampsia, and eclampsia are significantly higher in those having pre-medical conditions (Ghelichkhani et al., 2021). Interestingly, in one study, infected pregnant women, in their second and third trimesters without pre-existing medical conditions, were compared to their infected familial and household members, and found that the maternal outcomes were more severe (Hantoushzadeh et al., 2020). As for neonates born to infected mothers, in most of the studies, no vertical transmission was observed (Akbarian-Rad et al., 2021; Vaezi et al., 2021; Abedzadeh-Kalahroudi et al., 2021b). Scattered reports however reported the transmission of the SARS-CoV-2 virus from the infected mother to her newborn (Kamali Aghdam et al., 2020; Rashidian et al., 2020; Sattari et al., 2020; Zamaniyan et al., 2020; Abolhasan et al., 2021; Rabiei et al., 2021; Abedzadeh-Kalahroudi et al., 2021a). According to several studies (Alzamora et al., 2020; Penfield et al., 2020; Wu et al., 2020), it seems that vertical transmission increases when the mother experience a severe or critical course of the disease (Alipour et al., 2021; Farhadi et al., 2021; Vizheh et al., 2021). Viral load in the infected mother may be another contributing factor for the vertical transmission (Qiu et al., 2020; Fan et al., 2021). Neonatal death due to COVID-19 was only reported in four cases throughout the studies in Iran (Rashidian et al., 2020; Moeindarbary et al., 2021; Vizheh et al., 2021).

In the Kingdom of Saudi Arabia, very few studies explored the clinical impact of COVID-19 in pregnant women. One study found that the most common symptoms were cough, fever, and dyspnea (Al-Matary et al., 2021). The majority of infected pregnant women did not have comorbidities and experienced a mild or moderate disease (Alhamoud et al., 2020; Al-Matary et al., 2021; AlQurashi et al., 2021). A small proportion only, required intensive care and respiratory support as they developed symptoms of pneumonia; this finding was reported in pregnant women between 26 and 36 weeks of gestational age (Al-Matary et al., 2021). The low mortality rate and ICU admission reported from Iran and KSA are similar to the one observed in several studies worldwide conducted on pregnant women infected with SARS-CoV-2 (Allotey et al., 2020; Elshafeey et al., 2020; Juan et al., 2020; Smith et al., 2020). In pregnant women with COVID-19 in this country, vaginal delivery was more prevalent than the cesarian one (Algadeeb et al., 2020; Alhamoud et al., 2020; Al-Matary et al., 2021; AlQurashi et al., 2021). The most frequent adverse pregnancy outcomes were premature followed by fetal distress, and preeclampsia which occurred in pregnant women <37 weeks of gestational age (Al-Matary et al., 2021). No vertical transmission was reported (Al-Matary et al., 2021; AlQurashi et al., 2021). This is except for one case where the female infant experienced severe respiratory symptoms together with persistent pulmonary hypertension that led consequently to her death (Algadeeb et al., 2020).

In the United Arab Emirates, one study has shown that fever followed by myalgia, sore throat, cough, and shortness of breath are the most common symptoms observed in pregnant women infected with SARS-CoV-2. Similar to what is observed in other middle eastern countries, pregnant patients are mostly asymptomatic or have mild-to-moderate disease (Hazari et al., 2021). Severe COVID-19 illness, ICU admission, and intubation were observed in 10 infected pregnant women with prior comorbidities, of whom eight were in their third trimester, one in her second, and one in her first trimester. The mode of delivery was mostly performed based on obstetric indication, except for few cases where the lower segment cesarean section was done because of COVID-19 pneumonia (Hazari et al., 2021). Compared to non-pregnant women with COVID-19, infected pregnant women had more severe symptoms, ICU admissions, and complications. Furthermore, C-reactive protein and D-dimer levels were significantly higher in pregnant patients (Hazari et al., 2021). Fortunately, two studies have shown a minimal risk of vertical transmission as well as symptomatic infection of COVID-19 in neonates born to infected mothers (Hazari et al., 2021; Kaushal et al., 2021). In Qatar, two cases of infected pregnant women, one in her 26th week and one in her 32th week of gestation, were reported. Both had typical symptoms of COVID-19, i.e., fever, shortness of breath, cough, and sore throat. Cesarian section was performed for fetal safety with both newborns being negative for SARS-CoV-2 (Yaqoub et al., 2020; Khatib et al., 2021). Another interesting study conducted in Qatar, on 16 vaccinated and 370 unvaccinated pregnant women infected with SARS-CoV-2, found that 47% of the isolates were the Beta variant (B.1.351), 19% the alpha variant (B.1.1.7), and 34% were variants of unknown status. Eight severe cases and one critical case were recorded with all being in the unvaccinated group. Moreover, six were in their 1st trimester of pregnancy and two were in their second (Butt et al., 2021).

In Kuwait, one study reported that the most common symptoms of COVID-19 in pregnant women were fever and cough. The median gestational age of infected pregnant women in this study was 29 weeks with half of them being in their third trimester and the majority (up to 91%) having no pre-existing medical conditions. Vertical transmission was minimal with only two neonates out of 167, testing positive for SARS-CoV-2. One newborn was asymptomatic, while the other one required intensive care and was discharged later in a good condition (Ayed et al., 2020). In Bahrain, high incidence of COVID-19 was reported in pregnant women in their third trimester. Pregnant women were mostly asymptomatic with the symptomatic ones experiencing mild disease without the need for ICU admission. The complications by trimester of pregnancy were statistically significant for miscarriage where 25%–26% reported in the 1st trimester, 40% in the second, and 0% in the third one. No vertical transmission was observed (Sunder et al., 2022). In the Sultanate of Oman, it was shown that the risk of ICU admission, preterm labor, pre-eclampsia, and emergency lower segment C-section, in addition to thromboembolic complications, are increased in pregnant women with COVID-19. Similarly in this study, all neonates tested negative for the SARS-CoV-2 virus (Santhosh et al., 2021). In Egypt, only one study was conducted on pregnant women with COVID-19. In this latter, it was found that compared to non-pregnant women with COVID-19, pregnant patients tend to have more severe symptoms in the form of clinical pneumoniae, are more likely to be hospital and ICU admitted, need invasive mechanical ventilator, and experience severe outcomes of infection (BahaaEldin et al., 2021; Table 1).

In Jordan, one case of an infected pregnant woman in her third trimester, and who gave birth through C-section due to obstetric indication was reported. The newborn was SARS-CoV-2 negative and the mother was discharged later in a good general condition (AlZaghal et al., 2020). In Israel, scarce studies exist on the prognosis of COVID-19 in pregnant women. Two studies have shown that the most common symptom observed in this population is cough (Harel et al., 2021) and that the disease presents mostly as a mild or an asymptomatic one (Rottenstreich et al., 2021). The majority of pregnant women were in their third trimester at the time of COVID-19 diagnosis and delivery (Mohr-Sasson et al., 2020; Barber et al., 2021; Harel et al., 2021; Rottenstreich et al., 2021). Vaginal delivery was the most common, followed by C-section that was mainly performed based on obstetric indication and not due to the SARS-CoV-2 infection (Mohr-Sasson et al., 2020; Barber et al., 2021; Rottenstreich et al., 2021). Compared to non-pregnant patients, it was found in one study that the relative lymphocyte counts to white blood cells and the levels of WBC and absolute neutrophil counts are significantly reduced and increased in pregnant patients, respectively (Mohr-Sasson et al., 2020; Barber et al., 2021; Table 1). Furthermore, non-pregnant infected women are more likely to have chronic diseases and be hospitalized (Barber et al., 2021). With regard to neonates born to infected mothers, no vertical transmission was reported (Barber et al., 2021; Harel et al., 2021; Rottenstreich et al., 2021). In pregnant women, only one study was conducted to compare SARS-CoV-2 infected pregnant women vaccinated with a 1st dose of COVID-19 vaccine versus unvaccinated ones. This study was conducted in Israel and found that there is no notable difference in the rate of symptomatic infection, SARS-CoV-2-related hospitalization, or maternal complications such as pre-eclampsia, abortions, still birth, and maternal death between both groups (Goldshtein et al., 2021).

## Children

At the international context, many descriptive studies were conducted in children infected with SARS-CoV-2. However, little is known about this category in the middle eastern countries.

In Iran, the clinical outcome of SARS-CoV-2 infection in children is well described compared to other countries in the Middle East. It was found that the most common symptoms reported in children, i.e., <18 years old, are cough and dyspnea (Rabizadeh et al., 2020; Fahimzad et al., 2021) and fever and cough (Hosseninasab et al., 2020; Mahmoudi et al., 2020; Armin et al., 2021; Hoseinyazdi et al., 2021; Mamishi et al., 2021; Soleimani et al., 2021; Keshavarz Valian et al., 2022). Gastrointestinal symptoms including vomiting, nausea, and diarrhea were reported but to a lesser extent (Hosseninasab et al., 2020; Mamishi et al., 2020; Armin et al., 2021; Fahimzad et al., 2021; Pourakbari et al., 2021). Interestingly, neurological symptoms such as seizure, fatigue, and headache were also described (Cheraghali et al., 2021; Fahimzad et al., 2021). In one study, it was found that among hospitalized children with acute respiratory infection, those with COVID-19 had a higher frequency of respiratory distress and fever compared to those without (Hosseninasab et al., 2020). Furthermore, laboratory findings showed that hospitalized children with COVID-19 are more likely to have elevated inflammatory markers such as C-reactive protein, erythrocyte sedimentation rate (ESR), liver enzymes like alanine aminotransferase and aspartate aminotransferase (Armin et al., 2021; Hoseinyazdi et al., 2021; Mamishi et al., 2021); leukocytosis and leukopenia were also observed in one study (Armin et al., 2021). Prognosis of COVID-19 in Iranian children ranges from mild (Armin et al., 2021; Fahimzad et al., 2021) to severe disease (Mahmoudi et al., 2020; Ghazizadeh Esslami et al., 2021; Mamishi et al., 2021). In two studies, the SARS-CoV-2 infection manifested as a hyperinflammatory syndrome with multi-organ involvement similar to Kawasaki disease shock syndrome (Mamishi et al., 2021) and as MIS-C (Mamishi et al., 2020). In one study that reported severe infection in children, 51% of the patients had underlying medical conditions such as chronic kidney disease, neurological disorders, and others (Mahmoudi et al., 2020). In COVID-19 children, compared to those admitted to the general ward, ICU patients had more acute respiratory distress syndrome, shock, and acute cardiac injury as complications (Fahimzad et al., 2021). The mortality rate appears to be low in infected children of Iran (Hosseninasab et al., 2020; Armin et al., 2021; Hoseinyazdi et al., 2021; Mamishi et al., 2021; Pourakbari et al., 2021), ranging from 0 death (Esmaeili Dooki et al., 2020; Soleimani et al., 2021) to 11% (Mahmoudi et al., 2020; Mamishi et al., 2020; Armin et al., 2021). Interestingly, in one study the mortality rate was 20%, however, this was in children with underlying malignancies, namely acute lymphocytic and acute myeloid leukemia (Navaeian et al., 2021). The source of SARS-CoV-2 infection in the majority of the studies described in Iran was essentially contact history with suspected or confirmed family member with COVID-19 (Mahmoudi et al., 2020; Mamishi et al., 2020, 2021; Ghazizadeh Esslami et al., 2021; Navaeian et al., 2021). In one study, the source of infection was mainly unknown (Soleimani et al., 2021) and in another one, one case was infected during hospitalization (Mamishi et al., 2021).

In the Kingdom of Saudi Arabia, studies have shown a dominant benign prognosis of COVID-19 disease in children. A substantial portion were asymptomatic (Ahmad et al., 2021; Almuzaini et al., 2021; Hijazi et al., 2021) with this rate ranging from 27.9% (Mosalli et al., 2021) to 54.6% (Alharbi et al., 2021). Among symptomatic ones, the most common reported symptoms were fever, cough, runny nose, and shortness of breath (Harbi et al., 2020; Ahmad et al., 2021; Alharbi et al., 2021; Alshengeti et al., 2021; Mosalli et al., 2021; Shahin et al., 2021; AlGhamdi et al., 2022). Gastrointestinal symptoms were also described but to a lesser extent (Almoosa et al., 2020; Alharbi et al., 2021; Almuzaini et al., 2021; Alnajjar et al., 2021; Shahin et al., 2021). In some patients, the infection was first asymptomatic and presented later as a complicated course in the form of MIC-S (Almoosa et al., 2020; Al Qahtani et al., 2021 Shahin et al., 2021; Table 2). Among symptomatic ones, the rate of hospitalization ranged from 4.4% (Hijazi et al., 2021) to 9.6% (Alharbi et al., 2021). Similar to what was observed in Iran, it was found that pre-existing comorbidities, notably cardiac diseases, malignancies, and neurological and metabolic disorders, in addition to higher frequency of lower respiratory and gastrointestinal symptoms, are significantly associated with hospitalization in infected children (Alharbi et al., 2021); an observation that was also described in other studies (DeBiasi et al., 2020; Hendler et al., 2021; Woodruff et al., 2022). Moeller et al., conducted a multicenter study across 174 centers in Europe, found that children with chronic respiratory conditions might be at a higher risk of experiencing a severe form of COVID-19 (Moeller et al., 2020). In hospitalized COVID-19 children, the majority were admitted to the general ward and did not require intensive care (Alshengeti et al., 2021; Asseri et al., 2021; Almuzaini et al., 2021; Alnajjar et al., 2021; Hijazi et al., 2021; Kari et al., 2021b; AlGhamdi et al., 2022). On the other hand, those that needed intensive care had more frequent shortness of breath, higher absolute neutrophil counts and ferritin levels, and lower oxygen saturation, and albumin levels compared to those admitted to the general ward in the hospital (Asseri et al., 2021). Another study in KSA found that higher levels of creatinine values, leukocytes, and transaminases, in addition to worse renal functions, are significantly associated with ICU admission (Alharbi et al., 2021). Furthermore, the length of hospital stay was significantly correlated with the presence of comorbidities, leucopenia (Kari et al., 2021b), high levels of absolute neutrophil count (Shahin et al., 2021) as well as inflammatory markers such as ferritin, D-dimer, ESR and CRP (Kari et al., 2021b; Shahin et al., 2021). Indeed, the laboratory findings showed that among hospitalized patients, lymphocytopenia and leukopenia, in addition to elevated levels of ferritin, D-dimer, and C-reactive proteins, were encountered (Almoosa et al., 2020; Alnajjar et al., 2021; Mosalli et al., 2021; Shahin et al., 2021). The mortality rate was very low in the infected children of this country, with this latter not exceeding four deaths among all reports (Almoosa et al., 2020; Alnajjar et al., 2021; Alshengeti et al., 2021; Asseri et al., 2021; Hijazi et al., 2021; Kari et al., 2021b; Mosalli et al., 2021; AlGhamdi et al., 2022). Interestingly, in one study, it was found that 21% of hospitalized children infected with SARS-CoV-2 developed acute kidney injury. Children with AKI had a higher number of comorbidities compared to those with normal kidney function. Furthermore, AKI was significantly associated with more frequent ICU admissions as well as mortality. Residual renal impairment upon discharge occurred in 9% of those with AKI. This latter was influenced significantly by the presence of comorbidities, hypoxia, hypotension, heart failure, acute respiratory distress syndrome, hypernatremia, and high CRP level (Kari et al., 2021a). Like other countries, the main source of infection in Saudi children was family members, notably the father or the mother (Alnajjar et al., 2021; Shahin et al., 2021).

**Table 2:** Atypical presentations of COVID-19 in children in the Middle East.

**Country**	**Disease presentation**	**Comorbidities**	**COVID-19 Status**	**Most common symptoms**	**Laboratory findings**	**Hospitalization**	**Survival**	**Reference**
KSA	1 MIS-C		Laboratory confirmed COVID-19			PICU	Death^**^	Alshengeti et al., 2021
	1 MIS-C	Chediak-Higashi syndrome	Admitted with COVID-19			Hospitalized	Died	Mosalli et al., 2021
	1 MIS-C		Asymptomatic for 3 weeks after COVID-19 and Positive RT-PCR SARS-CoV-2	Fever, cough, shock, rash, pleural effusion	Lymphocytopenia, high inflammatory markers, notably CRP and ferritin	PICU	Recovered	Al Qahtani et al., 2021
	8 MIS-C		Laboratory confirmed COVID-19 infection			Hospitalized^**^	1 death	Asseri et al., 2021
	3 MIS-C		Confirmed COVID-19 infection			All PICU	All died	Kari et al., 2021b
	13 MIS-C		Confirmed Diagnosis of COVID-19			Hospitalized^**^	Death^**^	Kari et al. (2021b)
	5 MIS-C	Healthy	All positive for SARS-CoV-2 IgG and IgM, Infected with COVID-19 4–6 weeks before presentation	Fever, distress, hypoxia, malaise, dehydration, skin rash, dry lips, abdominal pain, and variable tachycardia		All ICU	All Recovered	Shahin et al., 2021
	5 MIS-C	1 Metabolic, 1 Neurologic (both in MIS-C)	Positive for SARS-CoV-2		All MIS-C elevated BNP and ferritin	All PICU	2 MIS-C death	Alharbi et al., 2021
	1 KD						4 Recovered	
	2 MIS-C	Medically free	Positive for SARS-CoV-2			All PICU	1 Death	AlGhamdi et al., 2022
	10 MIS-C	G6PD deficient, known T1DM,	Prior COVID-19 infection and exposure 14–31 days ago	Fever, abdominal pain and gastrointestinal symptoms	All acute anemia secondary to MIS-C	All PICU	2 Death	Almoosa et al., 2020
		and sickle cell trait			and hypoalbuminemia			
					Elevated inflammatory markers: either ESR or CRP			
Bahrain	1 HSP		Recent recovery (37 days ago) from COVID-19, Positive for SARS-CoV-2 RT-PCR	1-day history of rash in his lower limbs, and skin lesions	All normal	Not needed	Recovered	AlGhoozi and AlKhayyat, 2021
Egypt	4 MIS-C		Laboratory confirmed COVID-19.	Fever, cough, tachypnea, bilateral conjunctival injection, diffuse erythematous maculopapular rash, strawberry tongue.	Lymphopenia, thrombocytopenia, increased D-dimer, ferritin, and LDH levels, Mild increase in AST and ALT,	All Hospitalized	Death^**^	Saleh et al., 2021
	5 Acute Pancreatitis			Epigastric pain radiating to the back and associated ith fever, nausea, vomiting, and diarrhea	Elevated lipase and amylase, mild increase in AST and ALT			
	5 Deep venous thrombosis			Fever, mild dry cough, swelling, pain, warmth, and redness in left leg or right leg, or both legs.	Elevated median value of D-dimer			
	4 MIS-C	1 T1D, 3 newly diagnosed diabetes	All either SARS-CoV-2-positive PCR or positive antibody testing.	Persistent fever	Elevated CRP, D-dimer, ferritin, and LDH	All ICU		Sherif et al., 2021
Iran	2 similar KD shock syndrome^*^		Positive rRT-PCR test for SARS-CoV-2	Fever, cough, abdominal pain, maculopapular rash and conjunctivitis	Hypoalbuminemia and elevated CRP and D-Dimer levels	All hospitalized	Recovered	Mamishi et al., 2021
	45 MIS-C	ALL, CKD, seizure disorder, cerebral palsy, CVD, and Budd–Chiari syndrome	Positive of SARS-CoV-2 rRT-PCR or antibody assay.	Fever, abdominal pain, nausea/ vomiting, mucocutaneous rash, and conjunctivitis	Elevated ESR and CRP Hypoalbuminemia, and hyponatremia	All Hospitalized	5 Deaths	Mamishi et al., 2020
Iraq	31 MIS-C		80.6% positive for IgG, 13.3% positive for IgM	Fever and rash	High ESR, CRP, serum ferritin, and D-dimer levels	ICU^**^	Death^**^	Salih et al., 2022
Oman	5 MIS-C		All positive SARS-CoV-2 PCR and 3 positive COVID-19 IgG		Anemia		Recovered	Al Yazidi et al., 2021
Qatar	7 MIS-C		Two previous positive RT-qPCR for SARS-CoV-2	Fever, rash, and gastrointestinal symptoms	High CRP, procalcitonin, and ferritin	5 PICU	All recovered	Hasan et al., 2021
			At presentation, 1 positive NPS, 2/4 initially negative		Levels, and deranged coagulation profile			
			but later became positive COVID-19 serology					

MIS-C, Multisystem Inflammatory Syndrome in Children; KD, Kawasaki disease; HSP, Henoch-Schonlein purpura; ALL, Acute lymphocytic leukemia; ICU, intensive care unit; PICU, pediatric intensive care unit; CRP, C reactive protein; ESR, erythrocyte sedimentation rate; NPS, nasopharyngeal sample; AST, aspartate aminotransferase; ALT, alanine aminotransferase; LDH, lactate dehydrogenase; BNP, Brain natriuretic peptide.

* Two hyperinflammatory syndrome with multi-organ involvement.

** The number was not precised in the paper.

Al-Fraij et al. conducted a study both in KSA and Kuwait, where symptomatic children infected with SARS-CoV-2 and admitted to the ICU were explored. It was found that fever and cough were the most common symptoms at the time of ICU admission. The common cause of ICU admission was respiratory failure. On the other hand, low platelet counts, high procalcitonin, circulatory compromise, and the presence of comorbidities, such as hematological malignancies and neurological disorders, were associated with death in this group of population (Alfraij et al., 2021).

In Qatar, only two studies were conducted on Children with COVID-19. One included seven children who fulfilled the criteria of MIS-C according to WHO. The most common symptoms were fever, rash and gastrointestinal symptoms, and to a lesser extent upper respiratory tract ones. All cases included in the aforementioned study had high levels of procalcitonin, CRP, and ferritin levels, in addition to deranged coagulation profile. Five children required intensive care for inotropic support who later on were discharged in good condition (Hasan et al., 2021; Table 2). Notably, all children tested positive for COVID-19 antibodies, and only one tested positive by PCR test from nasopharyngeal swab; supporting thus the post-infectious nature of the MIS-C disease in this group (Hasan et al., 2021). The other study in Qatar was a case report of an 8-month-old infant with newly diagnosed diabetes (Soliman et al., 2020a). In Oman, the most common symptoms among symptomatic children infected with SARS-CoV-2 were fever (Alqayoudhi et al., 2021) followed by other respiratory and gastrointestinal symptoms (Al Yazidi et al., 2021). Hospitalized as well as critically ill patients were more likely to have pre-existing medical conditions. Moreover, intensive care was independently associated with leukocytosis and elevated C-reactive protein levels (Al Yazidi et al., 2021). No mortality was recorded (Al Yazidi et al., 2021; Al Sabahi et al., 2021; Alqayoudhi et al., 2021). As seen in other countries, family members were the main source of infection (Al Yazidi et al., 2021). Interestingly, in one study, it was found that 50 out of 1,026 children with COVID-19 were able to transmit the virus to 107 patients including 86 adults and 21 children (Alqayoudhi et al., 2021). In the UAE, one study found that the most common symptoms in infected children were fever, cough, and rhinorrhea. Patients presented with mild-to-moderate disease with no severe symptoms or mortality being reported. Furthermore, high levels of CRP and lactate dehydrogenase were significantly associated with symptomatic children. History of contact with family or household member was reported in the majority of infected patients (Ennab et al., 2021). In Iraq, Salih et al., found that fever was the common symptom detected in children with COVID-19. In this study, it was found that 62% of the cases showed signs of severe disease in the form of MIS-C. Around one-third required ICU admission with the majority of these being among MIS-C patients. Mortality was related to primary COVID-19 infection and to MIS-C cases (Salih et al., 2022). Another study reported the first case of delta variant B.1.617.2 in Iraq which was detected in a 6 years old female who presented at the hospital with fever, headache, severe abdominal pain, vomiting, and diarrhea. She was successfully treated and was discharged from the hospital in good condition (Essa et al., 2021). In Bahrain, AlGhoozi et al. reported a case where SARS-CoV-2 infection was possibly the trigger of Henoch-Schonlein purpura in a 4 years old boy (AlGhoozi and AlKhayyat, 2021).

In Egypt, fever, dry cough, polypnea, fatigue, headache, and shock were among the most common symptoms observed in children with COVID-19 (Shafiek et al., 2021; Saleh et al., 2021). Interestingly, in one study, in a subset of patients, the disease manifested by atypical presentations including deep venous thrombosis, acute pancreatitis, and MIS-C (Saleh et al., 2021; Table 2). Higher levels of D-dimer were significantly associated with disease severity (Shafiek et al., 2021; Saleh et al., 2021). The mortality rate was 5.3%, with these constituting 20.4% of the severe cases (Saleh et al., 2021). Definite contact with a SARS-CoV-2 infected family member was the main source of infection (Shafiek et al., 2021; Saleh et al., 2021). Sherif et al. conducted a study on infected children with type 1 diabetes and found that all four cases required ICU admission with the common symptoms being fever with respiratory or gastrointestinal ones. Three of the four cases presented with an MIS-C-like picture (Sherif et al., 2021). On the other hand, studies conducted on cancer children with COVID-19 showed that these can be either asymptomatic (Ebeid et al., 2021) or symptomatic with mainly mild-to-moderate form of the illness (Hammad et al., 2021; Hamdy et al., 2021). The mortality rate was as low as 6.5% after the exclusion of septicemia and cancer progression in one study (Hammad et al., 2021) and almost the half in another one (Hamdy et al., 2021). Interestingly, in both of the aforementioned studies, the poor outcome was mainly observed in those with hematological malignancies. In a study conducted in the United Kingdom, it was found that patients with hematological malignancies are at an increased risk of critical SARS-CoV-2 infection (Lee et al., 2020). On the contrary, another study in the US did not find an increased mortality in this type of cancer patients (Kuderer et al., 2020). Whether the effect observed is a true impact of COVID-19 or is due to the higher chemotherapy intensity given in these patients, should be more investigated in future studies.

In Israel, fever and cough were the most common symptoms observed in symptomatic children infected with SARS-CoV-2 (Ben-Shimol et al., 2021; Shapiro Ben David et al., 2021). Seizures in patients with prior neurological disorder were also reported (Kurd et al., 2021). Meyer et al., reported a case of an 8-month-old infant who contracted severe gastrointestinal symptoms, mild carditis, and significant hypoalbuminemia, following an asymptomatic infection with SARS-CoV-2 (Orlanski-Meyer et al., 2020). COVID-19 prognosis in Israeli children appears to be mainly mild (Ben-Shimol et al., 2021; Shapiro Ben David et al., 2021). In one study, pediatric inflammatory multisystem syndrome (PMIS) was detected in a subset of patients. Mechanical ventilation, intensive care in addition to lymphopenia, higher rates of neutrophils/lymphocytes, and elevated CRP and troponin levels were most common among PMIS patients compared to those with mild and moderate/severe disease (Ben-Shimol et al., 2021). No mortality was recorded. In one study that included 1,032 children with COVID-19, it was found that contact with a confirmed case was the most common source of infection followed by school exposure and unknown source (Shapiro Ben David et al., 2021). Among those who acquired the infection *via* a confirmed case, the majority were throughout infected parents. On the other hand, those who got the infection from school were mainly asymptomatic and were detected *via* contact tracing (Shapiro Ben David et al., 2021). The role of children in the transmission of SARS-CoV-2 appears to be less prominent than the one of adults (Dattner et al., 2021). Children cases are mainly secondary rather than primary (Shapiro Ben David et al., 2021). Only one study conducted by Somekh et al., explored the prognosis of COVID-19 in children in two different periods where the former SARS-CoV-2 variants (mainly GH and GR clades) were circulating in Israel versus when the B.1.1.7 variant was introduced. Results revealed a lower rate of hospitalization when the B.1.1.7 was introduced; however, the percentage of hospitalized children with poor outcomes, i.e., severe illness and/or death was not different between the two periods (Somekh et al., 2021). This implies that although B.1.1.7 appear to be more contagious but is not necessarily more severe, similar with what has been observed in other reports (Frampton et al., 2021; Galloway et al., 2021). Last but not least, in Jordan, the rate of asymptomatic vs. symptomatic children with COVID-19 was almost half, half. The most prominent symptoms were nasal congestion, generalized malaise, and headache. All children had infected parents (Kilani et al., 2021). In Lebanon, only one case of COVID-19 in children was reported (Mansour et al., 2020).

The favorable outcome of COVID-19 seen in children in the middle eastern population is similar to the one reported in other worldwide regions including Europe and China (Götzinger et al., 2020; Badal et al., 2021). Lower expression of angiotensin-converting enzyme 2 in children, differences in the immune system status as well as lower frequency of underlying medical conditions in children compared to adults has been suggested to play a role in the mild form of the COVID-19 illness in this population (Zimmerman et al., 2013; Falahi et al., 2021).

## Conclusion

**Figure 1:**
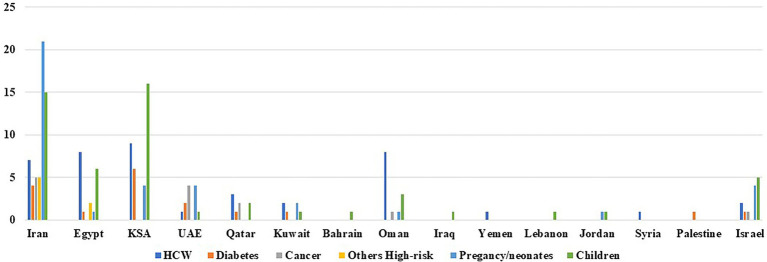
Number of published papers in each category per country in the Middle East.

In this review, we have highlighted several important issues regarding SARS-CoV-2 infection in the middle eastern region. First, there is a substantial proportion of asymptomatic carriage of among healthcare workers. This necessitates the implementation of a routine-based screening, not based on the presence of symptoms solely. This is in order to limit the transmission of the virus from the asymptomatic healthcare personnel to other patients, colleagues, or community members. This measure should be however tailored based on each country’s resource capacity. This is especially relevant in the Levant region, where financial and political crises are still ongoing. In fact, this is manifested in this review by the scarcity of studies exploring the prognosis of COVID-19 in different population categories in this region of the Middle East (Figure 1). High-risk patients appear to be at a higher risk of increased morbidity and mortality from SARS-CoV-2 infection compared to the general population (Table 1). Studies aimed to perform risk stratification of these high-risk patients are of paramount importance in the region. In fact, highlighting the clinical biomarkers during infection in these categories can be useful in the early suspicion of the disease, predicting clinical outcome, framing hospital and ICU admission/discharge, risk stratification; in addition to rationalizing therapies and assessing patients’ response to it. Unfortunately, enough data on which biomarkers can be used is still lacking in the Middle East. This also applies to infected pregnant women with their neonates. The vertical transmission of SARS-CoV-2 from the mother to the neonate is still controversial. Culturing of amniotic fluid, umbilical cord blood, in addition to neonatal nasopharyngeal swab is essential to explore this hypothesis. This should be done aseptically and at several time points after delivery. Last but not least, for children, although few studies were conducted; the current knowledge shows that SARS-CoV-2 infection is manifested as a benign form. This is not to omit, that physicians should be at the same time aware of the atypical presentations of COVID-19 in this group of population (Table 2). It is important to note also that the findings of this review are mainly based on studies conducted on unvaccinated people. The role of vaccine, viral load, variant of concerns, antiviral treatment in the prognosis and spread of COVID-19 in the categories described in this review should be addressed in future studies. This is in order to know the current true impact of this disease on different patients’ categories in the middle eastern region.

## Author contributions

All authors conceived and designed the review. ID wrote the manuscript. WA corrected the manuscript. All authors contributed to manuscript revision, and approved the final version.

## Conflict of interest

The authors declare that the research was conducted in the absence of any commercial or financial relationships that could be construed as a potential conflict of interest.

## Publisher’s note

All claims expressed in this article are solely those of the authors and do not necessarily represent those of their affiliated organizations, or those of the publisher, the editors and the reviewers. Any product that may be evaluated in this article, or claim that may be made by its manufacturer, is not guaranteed or endorsed by the publisher.
